# The effect of diabetes on burn patients: a retrospective cohort study

**DOI:** 10.1186/s13054-019-2328-6

**Published:** 2019-01-28

**Authors:** Reinhard Dolp, Sarah Rehou, Ruxandra Pinto, Rachel Trister, Marc G. Jeschke

**Affiliations:** 1 Sunnybrook Research Institute, Toronto, Ontario Canada; 2 Institute of Medical Science, Faculty of Medicine, University of Toronto, Toronto, Ontario Canada; 3 Ross Tilley Burn Centre, Sunnybrook Health Sciences Centre, Toronto, Ontario Canada; 4 Department of Critical Care Medicine, Sunnybrook Health Sciences Centre, Toronto, Ontario Canada; 5 Division of Plastic and Reconstructive Surgery, Department of Surgery, Faculty of Medicine, University of Toronto, Toronto, Ontario Canada; 6 Department of Immunology, Faculty of Medicine, University of Toronto, Toronto, Ontario Canada

**Keywords:** Burns, Thermal injury, Hyperglycemia, Blood glucose, Diabetes mellitus

## Abstract

**Background:**

Hyperglycemia during the acute phase after burn is associated with increased morbidity and mortality. There is little knowledge regarding the effect of pre-existing hyperglycemia in the form of diabetes on the outcomes after severe burns. The objective is to determine the impact of diabetes on clinical outcomes after burns.

**Methods:**

Single-center cohort study where adult diabetic (*n* = 76) and non-diabetic (*n* = 1186) burn patients admitted between 2006 and 2016 were included. Diabetic patients were stratified into those with well-controlled diabetes (*n* = 24) and poorly controlled diabetes (*n* = 33) using a HbA1c of 7% as a cutoff; additionally, diabetics were divided into well-controlled glycemia (*n* = 47) and poorly controlled glycemia (*n* = 22) based on daily blood glucose measurements during hospitalization.

**Results:**

On univariate analysis, diabetics had a significantly increased median length of stay per percent total body surface area burn (2.1 vs. 1.6 days; *p* = 0.0026) and a greater number of overall morbidity (1.39 ± 1.63 vs. 0.8 ± 1.24; *p* = 0.001). After adjustment for patient characteristics, diabetics were associated with significantly increased total morbidity (RR 1.5; 95% CI 1.1–1.9). At discharge, almost two thirds of diabetics needed an escalation of anti-diabetic medication and a quarter had newly developed insulin dependency. There were no differences in morbidity or mortality in the diabetic subgroups.

**Conclusions:**

Diabetics had a longer hospitalization and increased morbidity, regardless of the quality of their anti-diabetic therapy prior to injury. Additionally, diabetes in burn patients is associated with an increased risk of total morbidity.

**Electronic supplementary material:**

The online version of this article (10.1186/s13054-019-2328-6) contains supplementary material, which is available to authorized users.

## Introduction

Incidence and prevalence of diabetes have a seemingly unstoppable upward trend, already affecting approximately 9% of the USA (30.3 million Americans) and Canada (3.4 million Canadians) population [1, 2]. This makes diabetes one of the most common premorbid conditions for hospitalized patients.

A pre-existing condition such as diabetes greatly affects the body’s ability to cope with stress and is associated with glucose-related cell, end organ, and vascular damage and worsens clinical outcomes in hospitalized patients [3]. It is known, for example, that the constant exposure to hyperglycemia damages immune cells such as neutrophils leaving diabetics already at high risk for infections [4], one of the most feared complications in burn patients [5]. Chronic high blood glucose levels do not only increase the risk of patients to develop severe complications, but they also affect their ability to quickly recover after traumatic events such as burns. Diabetes leads to neuronal and axonal damage via inflammation that can affect every single nerve causing autonomic neuropathies such as extensive gastroparesis or severe hypotension further inhibiting fast rehabilitation and recovery [6]. It was already shown that diabetics overall have a higher rate of complications leading to a longer hospital stay and need more procedures such as fasciotomies and amputations [7, 8].

Burns cause acute stress-induced hyperglycemia which is known to increase mortality and morbidity [9, 10]. In the early phase after burn injury, hyperglycemia occurs due to decreased tissue extraction of glucose combined with increased glucose production and release caused by excessive secretion of stress hormones such as cortisol and epinephrine [11, 12]. Controlling hyperglycemia leads to an improvement in morbidity and mortality [13–15].

The exact role of pre-existing diabetes in burn patients is unclear with no knowledge about the impact of diabetic therapy prior to injury on outcomes after burn injury. The objectives and hypotheses of this study were as follows: First, to determine the role of diabetes in the outcome after burn (study A—Diabetics vs. non-diabetics). We hypothesized that diabetic patients have a significantly increased morbidity with a longer hospital stay. Second, to elucidate whether diabetes, in general, is a risk factor for poor outcomes after burn injury or if adverse outcomes are exclusively associated with diabetes that is poorly controlled (HbA1c > 7% at the time of admission). We expected that poorly controlled diabetes before burn results in worst clinical outcomes after burn compared to well-controlled diabetes (study B—Well vs. poorly controlled diabetes before burn). As a measure for the quality/success of diabetes control, we used the standard parameter glycated hemoglobin (HbA1c) that reflects hyperglycemia over the last 3 to 4 months [16, 17]. Third, to assess if diabetics that were hard to control in the acute setting after burn in terms of their blood glucose level had worse outcomes than diabetics that could be kept in the recommended blood sugar range (Study C - well vs. poorly controlled glycemia in hospital). We expected a higher rate of complications and mortality in diabetics whose blood sugar during the acute hospital phase after burn injury was hard to control, defined as a blood glucose level outside of the range 4.4 mmol/L (80 mg/dL) to 10 mmol/L (180 mg/dL) on more than two occasions after the first 7 days.

## Materials and methods

All adult admissions (*n* = 1262) to the Ross Tilley Burn Center from January 2006 to January 2016 were included. This protocol was approved by our institutional review board (#003-2011).

### Inclusion and exclusion criteria

#### Inclusion criteria

All patients ≥ 18 years admitted to the Ross Tilley Burn Center from January 2006 to January 2016 were included.

#### Exclusion Criteria

Death upon admission and decision not to treat due to burn injury severity and patients that participated in clinical trials to test new treatment modalities and therefore did not receive insulin as the standard of care treatment for glucose management were excluded.

### Study groups

#### Study A—Diabetics vs. non-diabetics

We determined the non-diabetic (*n* = 1186) and the diabetic patient cohort (*n* = 76) based on their medical history. We then looked at only diabetic burn patients in the following two study groups.

#### Study B—Well-controlled diabetes vs. poorly controlled diabetes

Diabetics were further divided due to the quality of their blood glucose/diabetes control prior to the burn trauma: poorly controlled diabetes (PCD) (*n* = 33, 58%) vs. well-controlled diabetes (WCD) (*n* = 24, 42%). The quality of chronic control was based on an HbA1c value above or below 7% at the time of admission. It is not a common practice to measure the HbA1c value at admission of a burn patient; therefore, only 57 out of the 76 diabetic burn patients had an HbA1c value and could be included in this section.

#### Study C—Well-controlled glycemia vs. poorly controlled glycemia

To investigate the effect of acute glucose control in hospitalized diabetics after burn, we divided the diabetic patient cohort into poorly controlled glycemia (PCG) (*n* = 22) and well-controlled glycemia (WCG) (*n* = 47). If blood glucose values after 7 days of admission exceeded 10 mmol/L (180 mg/dL) or fell below 4.4 mmol/L (80 mg/dL) on at least three occasions, the diabetic patient was defined with PCG. Diabetic patients that had no recorded blood glucose value 7 days after admission were excluded regardless of the reason why no blood glucose was measured. In total, we excluded seven patients.

### Demographics and outcome measurements

Patients’ demographics (age, sex, percent total body surface area (TBSA) burn, inhalation injury, and pre-existing diabetes) and outcomes (length of stay (LOS), length of stay per percent total body surface area (LOS/%TBSA) burn, complications, and mortality) were recorded, together with daily blood glucose values and insulin dosages.

Assessed complications were wound infections, bacteremia, sepsis, urinary tract infection (UTI), pneumonia, heart failure, renal failure, and respiratory failure. A wound was considered infected based on macroscopic appearance. Bacteremia was defined as a positive blood culture in the absence of fever. Sepsis was defined according to the ABA Guidelines (see Additional file 1 for the full criteria) [18]. Diagnosis of UTI included the macroscopic appearance of urine and positive urine culture. Pneumonia was defined as a pulmonary infiltrate, not attributable to cardiac causes, and combined with fever. Acute heart failure was diagnosed according to the current Canadian Cardiovascular Society Heart Failure Management Guidelines [19], acute renal failure according to the RIFLE criteria [20], and acute respiratory failure according to the American Thoracic Society [21].

Blood glucose values were gained via laboratory blood work. Point-of-care blood glucose measurements were excluded since recording and protocols in regard to frequency varied greatly over time. We first calculated a daily average blood glucose value for each patient (mean value), then we used those averages to calculate an overall average for the hospital stay of this patient. Insulin was assessed in the same way.

### Statistics

Continuous variables are summarized as means and standard deviations or medians and interquartile ranges (IQR), and differences between the groups were tested using *t* tests or the Wilcoxon rank-sum test. Discrete variables are reported as frequencies and percentage and compared between the groups using chi-square or Fisher’s exact test as appropriate.

Diabetic patients were matched one up to three non-diabetic patients on age (± 5 years), gender (exact), inhalation injury (exact), and TBSA (± 1%) using a greedy matching algorithm. We tested for the association between diabetes and morbidities within the matched group using conditional logistic regression for the binary outcomes and Poisson regression accounting for matching for the number of morbidities outcome. Due to a low death rate in the matched group and low event rate for mortality and morbidity in the diabetic subgroups adjusted analysis could not be performed.

The analysis was performed using SAS version 9.3 (SAS Institute Inc., Cary, NC, USA). All tests are two-sided and considered statistically significant at 5% significance level.

## Results

### Study A—Diabetics vs. non-diabetics

#### Demographics

A total of 1262 patients were included in this study, of which 76 were identified as diabetics. Table 1 shows the demographic data for the non-diabetic and the diabetic group. Patients in the diabetic group were significantly older (59.8 ± 16.8 years vs. 44.8 ± 17.3 years; *p* < 0.0001) but did not differ from the non-diabetic group in terms of TBSA, amount of second- and third-degree burns, and inhalation injury. Diabetic patients had a higher median LOS/%TBSA than non-diabetics (2.1 days vs. 1.6 days; *p* = 0.0027) and median LOS (19 days vs. 13 days; *p* < 0.0001) (see Table 1).

**Table 1:** Demographics and morbidity of diabetics vs. non-diabetics

	All	Diabetics	Non-diabetics	*p*
No. of patients	1262	76	1186	
Demographics				
Age, years, mean ± SD	45.7 ± 17.6	59.8 ± 16.8	44.8 ± 17.3	< 0.0001
Gender, male, no. (%)	901 (71.4)	51 (67.1)	850 (71.7)	0.4321
TBSA, %, median (IQR)	8 (3.5–16)	8 (5–14.8)	8 (3–16)	0.1391
TBSA 2nd degree, %, median (IQR)	4 (1–9.5)	4 (1–9.3)	4 (1–9.5)	0.9256
TBSA 3rd degree, %, median (IQR)	0.5 (0–5)	1.3 (0–7.8)	0.5 (0–5)	0.2317
LOS/%TBSA, median (IQR)	1.6 (1.0–2.8)	2.1 (1.5–3.3)	1.6 (0.9–2.7)	0.0026
LOS, median (IQR)	14 (7–22)	19 (13–26)	13 (7–21)	< 0.0001
Inhalation injury, no. (%)	196 (15.5)	14 (18.4)	182 (15.4)	0.4730
Death, no. (%)	44 (3.5)	5 (6.6)	39 (3.3)	0.1818
Morbidity				
Total morbidity, median (IQR)	0 (0–1)	1 (0–2)	0 (0–1)	0.00011
Wound infection, no. (%)	383 (30.3)	35 (46.1)	348 (29.3)	0.0021
Bacteremia, no. (%)	148 (11.7)	11 (14.5)	137 (11.5)	0.4427
Sepsis, no. (%)	104 (8.2)	11 (14.5)	93 (7.8)	0.0415
UTI, no. (%)	163 (12.9)	16 (21.1)	147 (12.4)	0.0291
Pneumonia, no. (%)	186 (14.7)	17 (22.4)	169 (14.3)	0.0529
Heart failure, no. (%)	15 (1.2)	4 (5.3)	11 (0.9)	0.01
Renal failure, no. (%)	34 (2.7)	5 (6.6)	29 (2.5)	0.0489
Respiratory failure, no. (%)	36 (2.9)	7 (9.2)	29 (2.5)	0.0044

*IQR* interquartile range, *LOS* length of stay, *SD* standard deviation, *TBSA* total body surface area, *UTI* urinary tract infection

#### Morbidity and mortality

**Fig. 1:**
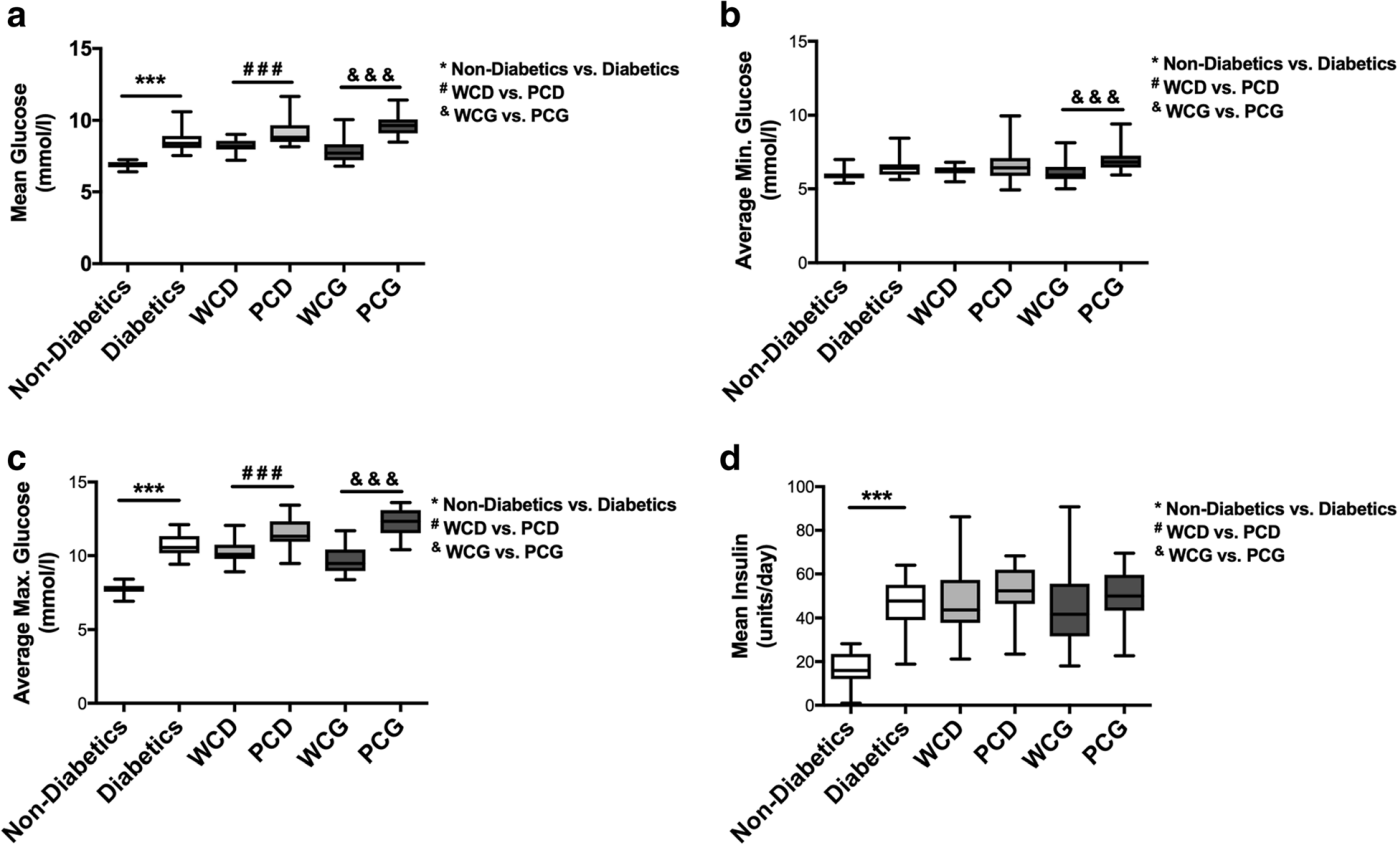
Mean glucose (**a**), average minimum glucose (**b**), average maximum glucose (**c**), and mean insulin values (**d**). PCD, poorly controlled diabetes, PCG, poorly controlled glycemia, WCD, well-controlled diabetes, WCG, well-controlled glycemia. ***, ###, and &&& indicate *p* < 0.001

Diabetic burn patients showed a significantly higher overall morbidity than non-diabetics (median (IQR) 1 (0–1) vs. 0 (0–1), *p* = 0.0001; Table 1 and Fig. 1). Especially, wound infections; sepsis; urinary tract infections; heart-, renal-, and respiratory failure were significantly higher in the diabetic cohort (46.1% vs. 29.3%, *p* = 0.0021; 14.4% vs. 11.5%, *p* = 0.0415; 21.1% vs. 12.4%; *p* = 0.0291; 5.3% vs. 0.9%, *p* = 0.01; 6.6% vs. 2.5%, *p* = 0.0489; 9.2% vs. 2.5%, *p* = 0.0044). The mortality in the diabetic group was higher than that in the non-diabetic group but was not statistically significant (diabetics vs. non-diabetics, 6.6% vs. 3.3%; *p* = 0.1818).

In the matched for age, inhalation injury, and percent TBSA burn cohort, there was an association between diabetics and a higher total morbidity than non-diabetics (RR 1.5; 95% CI 1.1–1.9) and respiratory failure (OR 4.8; 95% CI 1.1–20.0) (see Table 2). We did not compare the groups for mortality in the matched group due to the low number of deaths (six in both groups).

**Table 2:** Morbidity of matched diabetics (*n* = 68) to non-diabetics (*n* = 173)

Morbidity	Rate ratio	95% CI	*p*
Total morbidity	1.5	1.1–1.9	0.01
	Odds ratio		
Wound infection	1.4	0.8–2.4	0.2674
Sepsis	2.0	0.6–6.4	0.2549
UTI	0.8	0.4–1.8	0.6208
Pneumonia	1.9	0.8–4.5	0.1766
Renal failure	5.6	0.5–57.3	0.1461
Respiratory failure	4.8	1.1–20.0	0.0329

Matched one diabetic patient up to three non-diabetics by inhalation injury (exact), male (exact), age (± 5 years), and total body surface area (± 1%)

*CI* confidence interval, *UTI* urinary tract infection

#### Glucose level and insulin usage

Diabetic patients had a higher overall average blood glucose level during the hospital stay compared to the non-diabetic patients (8.5 ± 0.1 mmol/L vs. 6.9 ± 0.04 mmol/L, *p* < 0.0001). Figure 1 shows the overall daily minimum, maximum, and average blood glucose values for the different patient groups; Additional file 2 displays those values for each hospital day. Diabetic burn patients also showed an overall higher average need for insulin compared with the non-diabetic patients (36.1 ± 1.8 units vs. 16.3 ± 1.2 units, *p* < 0.0001) and required high dosages right after burn trauma, whereas non-diabetics showed a gradually increasing need for insulin during their hospitalization (see Fig. 1 and Additional file 3).

#### Diabetic medication

The majority of diabetic burn patients (63%) left the hospital with an escalated anti-diabetic medication, with a de novo insulin dependency of 24% and a new dependency for oral anti-diabetics of 11%. Twenty-six percent of the diabetic patients were discharged with either an increased dose or with an increased number of oral anti-diabetics. Thirteen percent of already insulin-dependent diabetics at the time of admission left the hospital with an increased dose of insulin (Additional file 4).

### Study B—WCD vs. PCD

#### Demographics

Based on their HbA1c value at the day of admission, 33 of the 76 diabetic patients were categorized as poorly controlled diabetics (PCD) and 24 as well-controlled diabetics (WCD).

Table 3 shows the demographic data for the PCD and the WCD group. Surprisingly, PCD were significantly younger than WCD (56.8 ± 16.5 vs. 67.0 ± 15.4; *p* = 0.0205). No difference could be found in their LOS/%TBSA, inhalation injury rate, or TBSA%.

**Table 3:** Demographics and morbidity of diabetics with well-controlled diabetes vs. poorly controlled diabetes

	All	WCD	PCD	*p*
No. of patients	57	24	33	
Demographics				
Age, years, mean ± SD	61.1 ± 16.7	67.0 ± 15.4	56.8 ± 16.5	0.0205
Gender, male, no. (%)	40 (70.2)	14 (58.3)	26 (78.8)	0.0956
TBSA, %, median (IQR)	8.5 (6–15)	9 (6–15.1)	8 (5–15)	0.6696
TBSA 2nd degree, %, median (IQR)	4 (1–8)	4.13 (0.3–9)	4 (1.5–8)	0.7895
TBSA 3rd degree, %, median (IQR)	2.5 (0–8.5)	2.63 (0–9.8)	2.5 (0–7)	0.8165
LOS/%TBSA, median (IQR)	2.13 (1.5–3.2)	2.2 (1.5–3.5)	1.9 (1.5–3)	0.6525
LOS, median (IQR)	20 (14–27)	24 (14.5–30.5)	18 (13–24)	0.0965
Inhalation injury, no. (%)	11 (19.3)	5 (20.8)	6 (18.2)	1.0000
Death, no. (%)	4 (7.0)	1 (4.2)	3 (9.1)	0.0956
Morbidity				
Total morbidity, median (IQR)	1 (0–2)	1 (0–2.5)	1 (0–1)	0.9528
Wound infection, no. (%)	27 (47.4)	8 (33.3)	19 (57.6)	0.0703
Bacteremia, no. (%)	9 (15.8)	6 (25.0)	3 (9.1)	0.1461
Sepsis, no. (%)	8 (14)	5 (20.8)	3 (9.1)	0.2612
UTI, no. (%)	11 (19.3)	5 (20.8)	6 (18.2)	1.0000
Pneumonia, no. (%)	12 (21.1)	7 (29.2)	5 (15.2)	0.2000
Heart failure, no. (%)	2 (3.5)	0 (0)	2 (6.1)	0.5038
Renal failure, no. (%)	4 (7)	1 (4.1)	3 (9.1)	0.6311
Respiratory failure, no. (%)	3 (5.3)	2 (8.3)	1 (3)	0.5669

*PCD* poorly controlled diabetes, *SD* standard deviation, *TBSA* total body surface area, *IQR* interquartile range, *UTI* urinary tract infection, *WCD* well-controlled diabetes

#### Morbidity and mortality

PCD patients showed a signal towards more wound site infections compared to the WCD group, but without reaching statistical significance (57.6% vs. 33.3%, *p* = 0.0703). No difference between the two groups could be found in the overall as well as in the individual morbidity (see Table 3). Due to the small sample size, no adjustment could be made for age, inhalation injury, and TBSA. Poor diabetes control resulted in a signal towards a higher mortality (9.1% vs. 4.2%, *p* = 0.361).

#### Glucose levels and insulin usage

PCD showed a higher overall average blood glucose level compared to WCD (9.1 ± 0.2 mmol/L vs. 8.2 ± 0.1 mmol/L, *p* < 0.0001). In addition, the PCD cohort had a higher daily max. glucose (11.6 ± 0.2 mmol/L vs. 10.3 ± 0.1 mmol/L, *p* < 0.0001; see Fig. 1). Poorly controlled diabetics received more insulin, but this was not statistically significant (52.8 ± 2.0 units for PCD vs. 46.4 ± 2.7 units for WCD, *p* = 0.056; see Fig. 1).

### Study C—WCG vs. PCG

#### Demographics

Under all diabetic burn patients, 47 were classified as WCG and 22 as PCG based on their daily blood glucose values. Table 4 shows the demographic data for the PCG and the WCG group. The PCG diabetics had a significantly higher median third-degree TBSA% patients (1 vs. 6, *p* = 0.0303) and a significantly longer median LOS (18 vs. 24; *p* = 0.0216) (see Table 4).

**Table 4:** Demographics and morbidity of diabetics with well-controlled glycemia vs. poorly controlled glycemia in hospital

	All	WCG	PCG	*p*
No. of patients	69	47	22	
Demographics				
Age, years, mean ± SD	60.1 ± 16.2	57.8 ± 14.9	65.2 ± 18.1	0.0707
Male, no. (%)	47 (68.1)	33 (70.2)	14 (63.6)	0.5849
TBSA, %, median (IQR)	9.5 (6–16)	7.5 (5–16)	13 (8–15)	0.2132
TBSA 2nd degree, %, median (IQR)	4 (1–9.5)	4.3 (1–9.5)	3.5 (0.5–9.5)	0.6064
TBSA 3rd degree, %, median (IQR)	2.5 (0–9)	1 (0–5)	6 (1–12)	0.0303
LOS/%TBSA, median (IQR)	2.1 (1.5–3.3)	2.3 (1.4–3.7)	1.8 (1.5–3)	0.5217
LOS, median (IQR)	19 (14–27)	18 (13–24)	24 (19–31)	0.0216
Inhalation injury, no. (%)	14 (20.3)	11 (23.4)	3 (13.6)	0.5229
Death, no. (%)	5 (7.3)	2 (4.3)	3 (13.6)	0.3176
Morbidity				
Total morbidity, median (IQR)	1 (0–3)	1 (0–3)	1 (0–4)	0.8416
Wound infection, no. (%)	34 (49.3)	25 (53.2)	9 (40.9)	0.3416
Bacteremia, no. (%)	11 (15.9)	7 (14.9)	4 (18.2)	0.7345
Sepsis, no. (%)	11 (15.9)	8 (17.0)	3 (13.6)	1.0000
UTI, no. (%)	15 (21.7)	10 (21.3)	5 (22.7)	1.0000
Pneumonia, no. (%)	17 (24.6)	10 (21.3)	7 (31.8)	0.3436
Heart failure, no. (%)	4 (5.8)	2 (4.3)	2 (9.1)	0.5874
Renal failure, no. (%)	5 (7.3)	2 (4.3)	3 (13.6)	0.3176
Respiratory failure, no. (%)	6 (8.7)	4 (8.5)	2 (9.1)	1.0000

*IQR* interquartile range, *LOS* length of stay, *PCG* poorly controlled glycemia, *SD* standard deviation, *TBSA* total body surface area, *UTI* urinary tract infection, *WCG* well-controlled glycemia

#### Morbidity and mortality

Interestingly, diabetics that were in the PCG group did not show statistically more morbidity than the WCG group. Due to the small sample size, no adjustment could be made for age, inhalation injury, and TBSA. Table 3 shows the overall as well as the individual morbidity for the two groups. Poor glucose control in diabetics while in hospital resulted in a clear signal towards a higher mortality but was not statistically significant (13.6% vs. 4.3%, *p* = 0.3176).

#### Glucose levels and insulin usage

Thirty-two percent (22 out of 69) of diabetic patients had glucose values > 10 mmol/L on more than 2 occasions, after day 7 of admission. PCG diabetics had a significantly higher overall, higher maximum, and higher minimum glucose values than WCG diabetics (9.6 ± 0.1 mmol/L vs. 7.8 ± 0.2 mmol/L, *p* < 0.0001; 12.2 ± 0.12 mmol/L vs. 9.7 ± 0.2 mmol/L, *p* < 0.0001; 6.0 ± 0.1 mmol/L vs. 6.9 ± 0.1 mmol/L, *p* < 0.0001; see Fig. 1). Patients in the PCG group received more insulin, but this was not statistically significant (46.1 ± 3.3 units for WCG vs. 50 ± 2.1 units for PCG, *p* = 0.339; see Fig. 1).

## Discussion

An estimate of the American Burn Association for 2016 concludes that approximately 486,000 burn injuries will need treatment, and 40,000 will have to be hospitalized [22]. With a projected increase in diabetes incidence to 165% by 2050 [23], we will see more burn patients with this condition, and to know its effect on the clinical outcome is paramount. Diabetes causes metabolic derangements, wound healing disorders, immune dysfunction, and vascular damage via glycosylation. Therefore, it seems logical to hypothesize that patients with a severe premorbid condition like diabetes, especially if poorly controlled, have a worse clinical outcome after superimposed critical diseases such as burns. Data for the effects of diabetes on the outcomes after burns are currently inconsistent or inconclusive; no data exists that evaluates the role of diabetes management prior to burn trauma.

Severe burns are associated with a high number of complications even in otherwise healthy individuals, with infection and sepsis being one of the major contributors to morbidity and mortality [5, 7]. The most common sources of infections in burn patients are the burn wounds itself, the gastrointestinal tract, the respiratory tract, and iatrogenic bacteremia [5]. Diabetes as a premorbid condition leads to an increased infection rate in burn patients [7, 24]. This study confirms those previous results. All diabetic patients displayed more infections than non-diabetics with sepsis, wound infections, and UTI. Although we did not determine the incidence of abdominal compartment syndrome in this study, it has become evident that in the pathogenesis of diabetic neuropathy, the role of the abdominal compartment is becoming increasingly more evident and concerning [25].

In the matched cohort for age, inhalation injury, and TBSA%, the overall morbidity and respiratory failure still remained significantly higher in the diabetic cohort. Burn patients in the PCD group showed a signal towards more wound infections, but due to the small sample size, the complications in the diabetic subgroups could not be adjusted to the age, inhalation injury, and TBSA. Diabetics whose blood glucose level was hard to control in the acute post-traumatic phase (PCG) did not display a higher complication rate than their well-controlled counterparts. This might be due to the fact that, even though their daily maximum exceeded 10 mmol/L (180 mg/dL) on more than two occasions after 7 days of admission, their daily average glucose value was still inside the recommended goal of < 10 mmol/L (180 mg/dL) and no patient had a severe hypoglycemia below 4.4 mmol/L (80 mg/dL). That is a reassuring finding confirming the efficacy of the current in-hospital glucose management since it shows that even those patients whose blood sugar is hard to control have no more adverse findings than their control group.

The diabetic cohort had a higher risk of organ failure, in this study as well as in the literature [26], with heart, renal, and respiratory failure being statistically significant. When adjusted for age, inhalation injury, and TBSA, only respiratory failure was statistically significant. It is to be expected that a higher diabetic patient cohort would lead to more significance. Diabetes is a known cause of end-organ damage which explains the higher susceptibility of organ failure in the diabetic patient group [26]. In addition, diabetic burn patients are older and have a higher number of respiratory and cardiac diseases at the time of admission [15]. Interestingly, those in the PCD group (HbA1c > 7%) did not show a higher overall complication rate than the WCD group (HbA1c < 7%). However, the HbA1c value only reflects the quality of the blood glucose treatment for the past 2 to 3 months. No definitive conclusion can be drawn about the long-term quality of diabetic treatment and the severity of pre-existing diabetes-associated damages in the evaluated patients.

Despite the increase in the overall morbidity in diabetic burn patients, mortality was not statistically different. This is in accordance with the current literature that also did not show statistically increased mortality in diabetic burn patients [7, 27–29]. The lack of a clear significant difference in mortality between the two groups could be explained simply by the low number (*n* = 76) of diabetes patients. Other explanatory models exist in the literature for the phenomenon that diabetes does not affect mortality in burns. The first model is that patients with severe diabetes-associated comorbidities do not survive the burn trauma, and therefore, only diabetic patients with sufficient physiological resources are assessed [30]. Another explanation might be that diabetes as a premorbid condition is outweighed by more severe factors such as TBSA [27]. The quality of hyperglycemia control in diabetic patients—before and after burn—seems to be an important variable in the mortality of diabetic burn patients. Diabetics with an HbA1C value > 7% at the time of admission (PCD) did display a higher mortality. The reduction of acute post-traumatic hyperglycemia is a key element in decreasing mortality and morbidity in burn patients [13–15]. Our data in the diabetic cohort shows a lower mortality in patients with a well-controlled blood glucose level in the hospital phase after burn trauma (WCG) compared with diabetic patients that had blood glucose levels which were hard to keep in the recommended goal of < 10 mmol/L (PCG).

A comparison of the diabetic medications at the time of discharge with the medications at the time of admission yielded an interesting observation. Not only does diabetes affect burns, but also does burn trauma affect diabetes—in the form of an escalation of anti-diabetic medication in almost two thirds of all diabetic patients. The fact that post-traumatic hyperglycemia is persistent for up to 6 weeks and insulin resistance for up to 3 years after burn trauma is known [31], there is no study evaluating how these changes affect diabetic patients and their anti-diabetic medication in the long term. Further studies need to determine if the burn-induced hyperglycemia leads to a faster progression of pre-existing diabetes or if those changes are mainly transient like in non-diabetic patients. Considering the high socioeconomic costs of diabetic treatment, this is essential in improving the prediction of costs and long-term outcome after burn trauma in diabetic patients.

While this study did include patients over a substantial time period, there were multiple limitations. Firstly, this is a single-center cohort study, and given its retrospective nature, the conclusions are limited to associations only. Secondly, the change in burn patient management, such as increased implementation of standardized protocols, over the 10-year period might have affected glucose control. A time effect is also the reason we excluded point-of-care tests, because of greater use of them in recent years. This means we may have missed some hypoglycemic or hyperglycemic events. However, glucose levels are not routinely measured multiple times, unless there is a clinical indication. Additionally, diabetic patients might also have other pre-existing comorbidities that could affect their hospital stay and clinical outcomes. We further recognize that by using blood glucose levels with a 7-day cutoff, there may be a survivorship bias. We would like to note that the seven diabetic patients that were excluded from the analysis of well-controlled acute hyperglycemia vs. poorly controlled acute hyperglycemia all survived. Lastly, we would like to acknowledge that blood glucose levels may not reveal the entire picture in the setting of post­injury critical illness and the metabolic reprogramming associated with recovery. Using the methods of Elrick et al. [32], Wilmore and colleagues [33] demonstrated that glucose flux was elevated two- to threefold above normal after burn injury indicating the hypermetabolic response of glucose flux. The authors subsequently showed that glucose flux fell to subnormal (unburned) levels in a group of burned patients with gram-negative bacteremia [34]. In the latter paper, the authors indicated that reduced hepatic glucose transport is compensated by reduced insulin secretion. Therefore, serum glucose most likely does not reveal the entire complex metabolic picture and certainly limit the use of blood glucose as a surrogate for altered glucose homeostasis. This is confirmed by the distinction between post­injury recovery hypermetabolism and post­septic metabolic derangement. Both of the latter states may lead to hyperglycemia, although through very different mechanisms. In fact, there is some indication that the post­burn hypermetabolic state, driven by high levels of catecholamines, may starkly increase the peripheral tissue glucose uptake, particularly in the absence of sepsis. It remains an unanswered question whether this is a contributor to the increased blood glucose variability observed in burn patients and also in our PCG subgroup analysis or whether these effects are correlated and have no direct effect at all. To better understand the glucose metabolism and the consequences of hyperglycemia in a burn recovery state or in a diabetic burn recovery state, much more cellular and granular metabolic work needs to be conducted.

## Conclusions

This study demonstrates, in a Canadian population, that burn patients with diabetes as a premorbid condition had poor clinical outcomes and a longer hospital stay. After the adjustment for patient characteristics and injury severity, diabetic patients are associated with increased total morbidity and respiratory failure.

## Additional files

Additional file 1: **Table S1.** American Burn Association Sepsis Criteria for adults. (DOCX 17 kb)

Additional file 2: Daily mean, minimum, and maximum glucose values for diabetics vs. non-diabetics (A), well-controlled vs. poorly controlled diabetes (B), and well-controlled vs. poorly controlled glycemia (C). PCD = poorly controlled diabetes, PCG = poorly controlled glycemia. WCD = well-controlled diabetes, WCG = well-controlled glycemia. (TIFF 278 kb)

Additional file 3: Daily mean insulin for diabetics vs. non-diabetic (A), well-controlled vs. poorly controlled diabetes (B), and well-controlled vs. poorly controlled glycemia (C). PCD = poorly controlled diabetes, PCG = poorly controlled glycemia. WCD = well-controlled diabetes, WCG = well-controlled glycemia. (TIFF 138 kb)

Additional file 4 Anti-diabetic medication usage at discharge from hospital. OA = oral antidiabetic. (TIFF 243 kb)
