# Supplementary material for: The effect of diabetes on burn patients: a retrospective cohort study
Source: Crit Care. 2019 Jan 28;23:28. doi: 10.1186/s13054-019-2328-6 (PMC6348623; doi:10.1186/s13054-019-2328-6)
Supplement: Supplementary file 1 — Table S1. American Burn Association Sepsis Criteria for adults. (DOCX 17 kb) [file 13054_2019_2328_MOESM1_ESM.docx]

| **Additional File Table 1.** American Burn Association Sepsis Criteria for Adults. |
| --- |
| **Sepsis should be considered when at least 3 of the following criteria are met:** |
| 1. Temperature: >39°C or <36.5°C 2. Progressive tachycardia: >110 beats per min 3. Progressive tachypnea: >25 breaths per minute not ventilated    - Minute ventilation >121 L/min ventilated 4. Thrombocytopenia (not applicable until 3 days after initial resuscitation): <100,000/μL 5. Hyperglycemia (in the absence of pre-existing diabetes mellitus)    - Untreated plasma glucose >200 mg/dL or equivalent mM/L 6. Insulin resistance example:    - >7 units of insulin/hour intravenous drip    - Significant resistance to insulin (>25% increase in insulin requirements over 24 hours) 7. Inability to continue enteral feedings for >24 hours    - Abdominal distension    - Enteral feeding intolerance (residuals > two times feeding rate)    - Uncontrollable diarrhea (>2,500 mL/day) |
| **Additionally, at least one of the following infections must be met:** |
| 1. Culture positive infection, or 2. Pathologic tissue source identified, or 3. Clinical response to antimicrobials |
| Table adapted from the American Burn Association consensus conference to define sepsis and infection in burns.[18] |
