# Supplementary figures and images for: The effect of diabetes on burn patients: a retrospective cohort study
Source: Crit Care. 2019 Jan 28;23:28. doi: 10.1186/s13054-019-2328-6 (PMC6348623; doi:10.1186/s13054-019-2328-6)

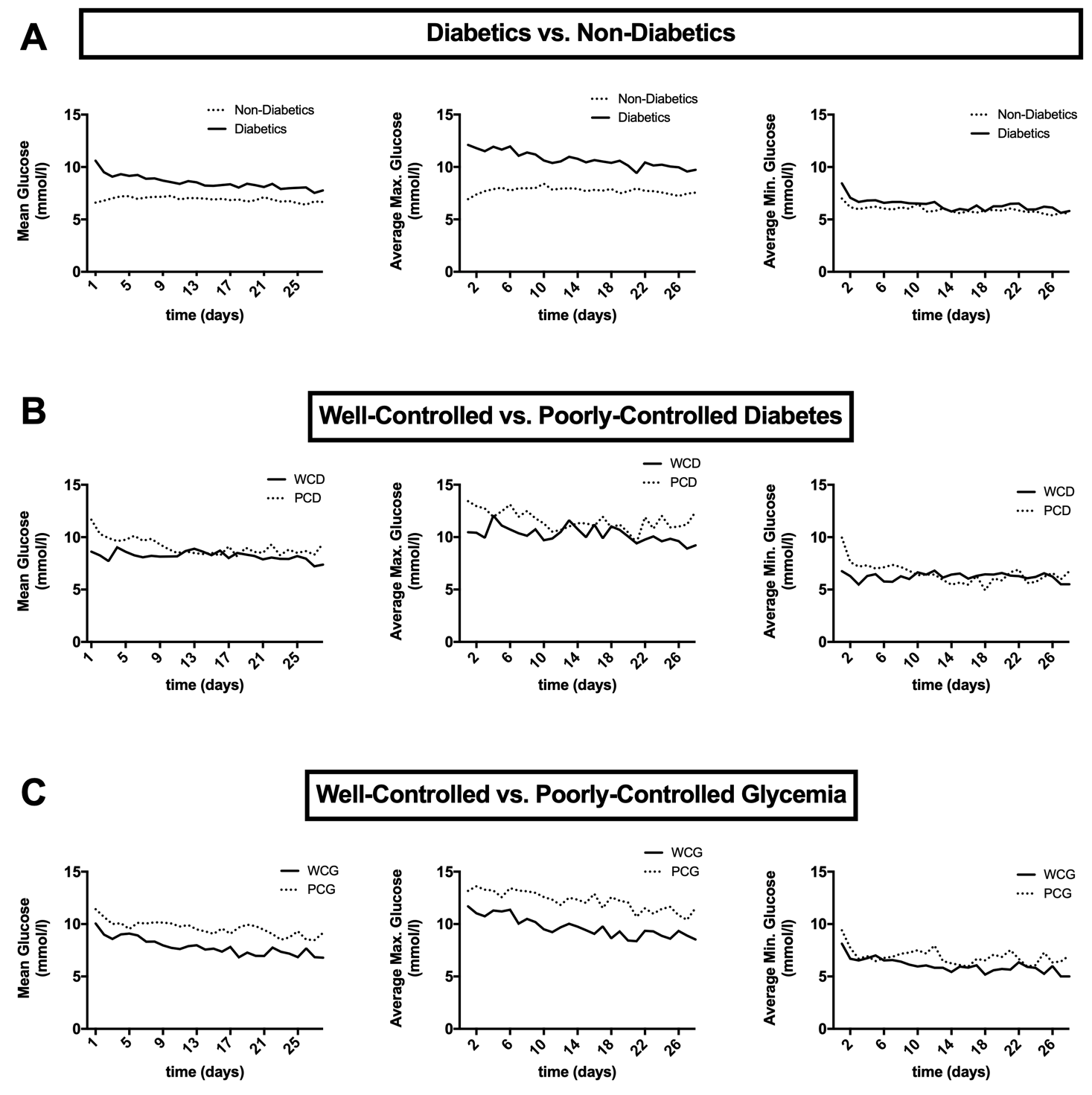

Supplement: Supplementary file 2 — Daily mean, minimum, and maximum glucose values for diabetics vs. non-diabetics (A), well-controlled vs. poorly controlled diabetes (B), and well-controlled vs. poorly controlled glycemia (C). PCD = poorly controlled diabetes, PCG = poorly controlled glycemia. WCD = well-controlled diabetes, WCG = well-controlled glycemia. (TIFF 278 kb) [file 13054_2019_2328_MOESM2_ESM.tiff]

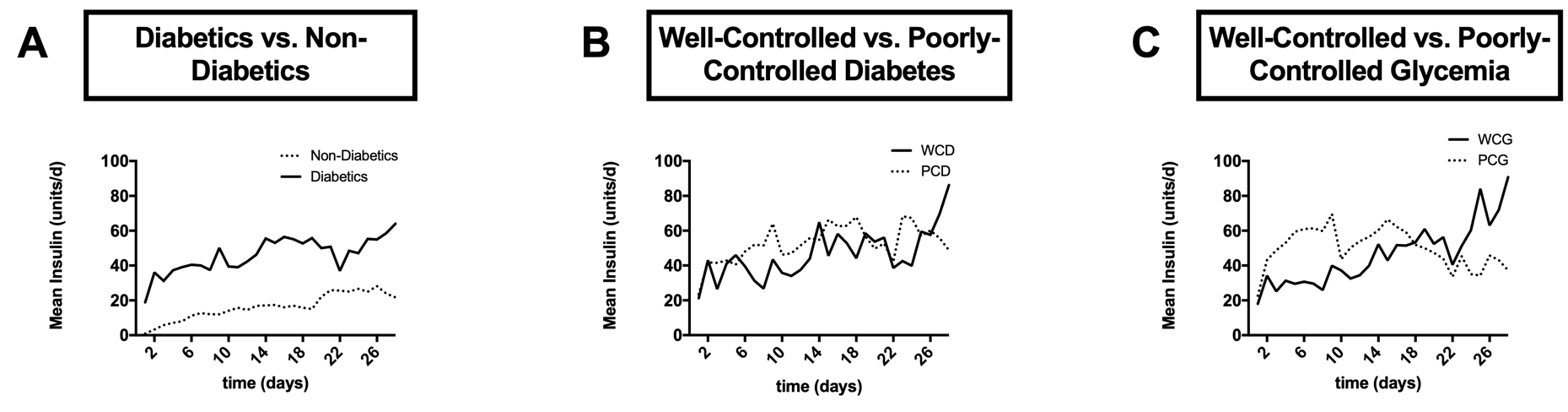

Supplement: Supplementary file 3 — Daily mean insulin for diabetics vs. non-diabetic (A), well-controlled vs. poorly controlled diabetes (B), and well-controlled vs. poorly controlled glycemia (C). PCD = poorly controlled diabetes, PCG = poorly controlled glycemia. WCD = well-controlled diabetes, WCG = well-controlled glycemia. (TIFF 138 kb) [file 13054_2019_2328_MOESM3_ESM.tiff]

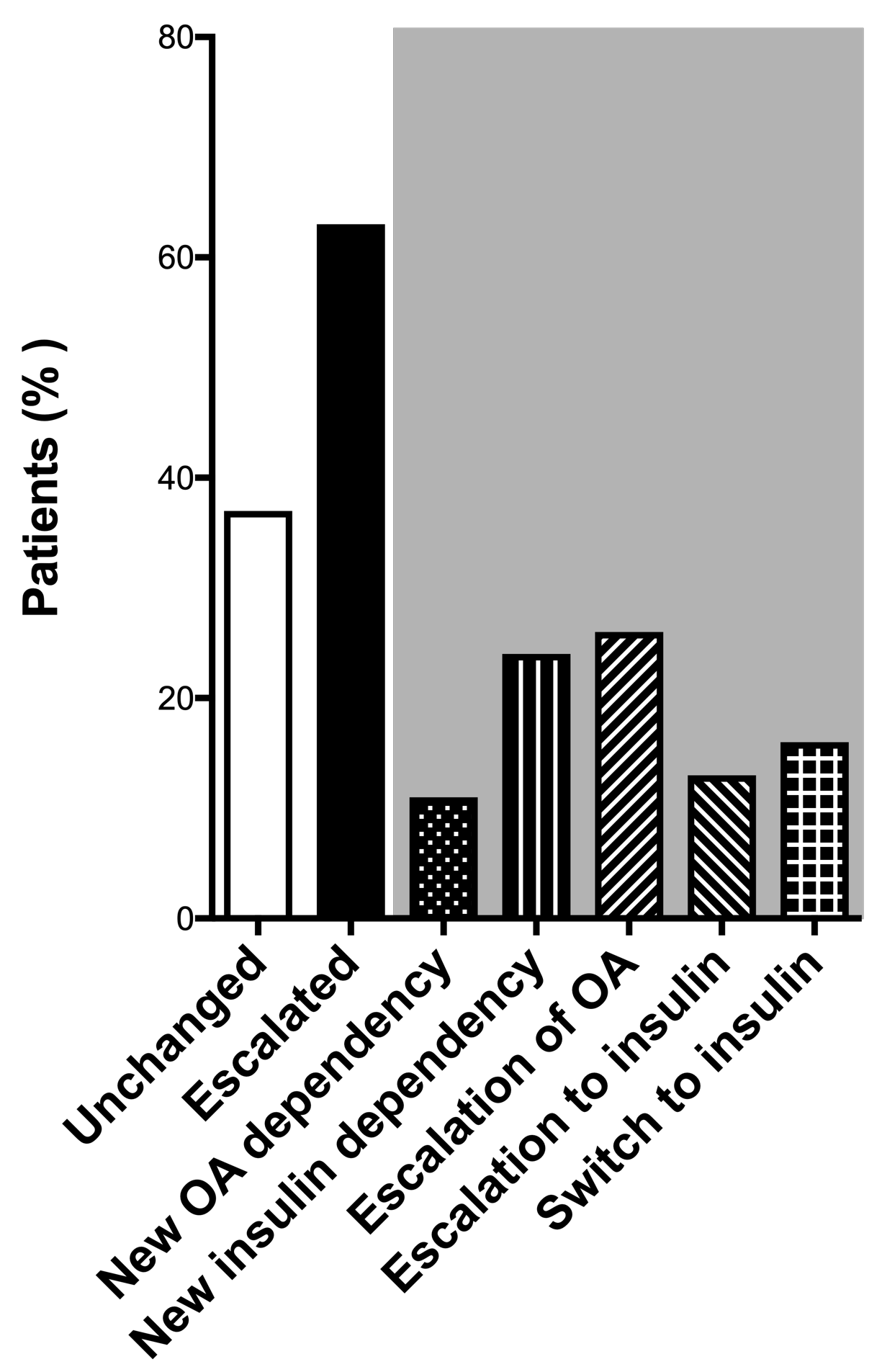

Supplement: Supplementary file 4 — Anti-diabetic medication usage at discharge from hospital. OA = oral antidiabetic. (TIFF 243 kb) [file 13054_2019_2328_MOESM4_ESM.tiff]
